# Sex differences in the association of postural control with indirect measures of body representations

**DOI:** 10.1038/s41598-022-07738-8

**Published:** 2022-03-16

**Authors:** Katrin H. Schulleri, Leif Johannsen, Youssef Michel, Dongheui Lee

**Affiliations:** 1 Department of Electrical and Computer Engineering (EI), Human-centered Assistive Robotics (HCR), Technical University of Munich (TUM), 80333 Munich, Germany; 2 Institute of Psychology, Cognitive and Experimental Psychology, RWTH Aachen University, 52066 Aachen, Germany; 3 Department of Sport and Health Sciences, Human Movement Science, Technical University of Munich (TUM), Munich, 80992 Germany; 4 Institute of Robotics and Mechatronics, German Aerospace Center (DLR), 82234 Weßling, Germany

**Keywords:** Human behaviour, Motor control, Sensorimotor processing, Somatosensory system, Psychology

## Abstract

Besides anthropometric variables, high-order body representations have been hypothesised to influence postural control. However, this has not been directly tested before. Moreover, some studies indicate that sex moderates the relationship of anthropometry and postural control. Therefore, as a proof of concept we investigated the association of body representations with postural control as well as the influence of participants’ sex/gender. Body image measures were assessed with a figural drawing task. Body schema was tested by a covert and an overt task. Body sway was measured during normal bipedal quiet standing with eyes closed (with/without neck extended). Statistical analysis consisted of hierarchical multiple linear regressions with the following regression steps: (1) sensory condition, (2) sex/gender, (3) age, (4) anthropometry, (5) body schema, (6) body image, (7) sex/gender-interactions. Across 36 subjects (19 females), body schema was significantly associated with body sway variability and open-loop control, in addition to commonly known influencing factors, such as sensory condition, gender, age and anthropometry. While in females, also body image dissatisfaction substantially was associated with postural control, this was not the case in males. Sex differences and possible causes why high-order body representations may influence concurrent sensorimotor control of body sway are discussed.

## Introduction

A specific domain of motor control, which is constantly and mainly unconsciously involved in daily life is, postural control. By maintaining the center of mass (CoM) within the base of support (BoS), it helps to stay upright and prevents from falling^1^, e.g. when standing, walking or also when performing sports. Postural control during quiet standing is influenced by several factors, such as age^2,3^, biological sex^4–6^, anthropometry^7–9^, stance condition or BoS^3^ and the available sensory channels^10^. When being involved in a secondary task or a suprapostural task, like often in everyday life, postural control further depends on the type and constraints of a task showing differences e.g. in the positional variability^11–14^, thus is task-specific. Often the functional postural control goal is to reduce sway variability to facilitate suprapostural task performance^11–14^ However, the postural control goal could also sometimes be to increase sway variability and perform exploratory movements to gain more information about the environment and the orientation of the own body in space^15–17^.

Since body sway during quiet standing is often simulated by a single inverted pendulum model^18,19^, one important factor influencing human body sway, from biomechanical point of view, is anthropometry, such as body height and body weight/mass^7,9^, as well as body morphology^4,20^. Even though the single inverted pendulum discards multi-joint movements compared to the double inverted pendulum model, which is commonly used to capture intra-personal coordination patterns between ankle and hip movements^21–24^, the single inverted pendulum model has been argued to functionally correctly capture body sway during quiet standing, using the ankle strategy^25^, in a simplified and practically acceptable way^18^.

Moreover, as mentioned before, sex differences have also been reported to affect body sway; whereby, females often show less body sway than males^4,6^. However, when body sway is normalised by body height or weight, previously observed sex differences disappear^9^, which indicate anthropometry mediating the influence of gender on body sway. In spite of that, sex differences have also been reported in the relationship of anthropometry and body sway^7^; thereby some studies have reported a greater influence of anthropometry on body sway in males than in females^7^, while others have reported the opposite^26^. The previously reported influencing factors, however, can only explain up to approximately 47% of the inter-individual variability in human body sway^3,8,26^.

Therefore, other factors might additionally play a role, such as eventually aspects of higher order representations. For example, in the work of Forghieri and colleagues^27^ when subjects saw a mirror image or their ideal model image during quiet standing, patients with eating disorders, who also showed a higher body dissatisfaction level, had a greater sway increase compared to the control group. Body dissatisfaction addresses one component of *body image*, which is defined as a conscious, persistent representation of our body size and shape^28^, and contains different components attributed to the own body shape, such as the physical appearance (e.g. BID: body image distortion) and emotional attitudes (e.g. BIDS: body image dissatisfaction)^29^. It is influenced by social and cultural expectations^30^. Moreover, body image dissatisfaction differs in gender^29^, whereby a greater dissatisfaction is observed among females compared to males. Consequently, disorders involving a distorted body image, are also more prevalent in females^29^. Thus, gender differences in the influence of anthropometry on body sway might be due to differences in the role of high-order body representations, such as the body image.

Another type of high-order body representations, that has been discussed to influence motor control, is the body schema^31^. The *body schema* can be described as an unconscious, dynamic, multi-referential, spatio-temporal and somatotopic real-time representation of our body’s metrics and configuration in space, which is action- and task-relevant^28,31^. In the context of postural control body schema is often referred to as an *internal model*^32–34^. Body sway can be generally described by two control mechanisms: the feed-forward and the feedback control^35,36^. The feed-forward control is often discussed to be based on an internal body representation of geometry, kinetics, verticality and reference frames inducing voluntary and anticipatory movements^32^. Respectively, the feed-forward component is attributed to the slow dynamics of body sway, accounting for most of the body sway^37^, whereas the fast dynamics represents the corrective feedback component^38^.

In everyday life, when physically interacting with the environment, the control system point of view on motor control involves internal representations of the own body and its immediate environment (e.g. the peripersonal space)^39^ in addition to the integration of diverse sensory modalities. Since our body schema receives continuous feedback from various sensory channels during overt movements, it is continuously updated by motor experience and given a current task (e.g. tool-use^40,41^); thus, it is discussed to improve with movement experience^32^, as well as with increased proprioceptive awareness/reliance or self-awareness, such as observed in dancers^42–44^. Moreover, mental simulation of an action has been shown to share central-nervous processes with real actions^45^, while not including movement-related sensory feedback. Consequently, the body schema is presumably needed to enable performing both overt (actual) and covert (simulations or motor imagery) movements/actions^46^ and is supposed to merge both motor cognition and motor control^31^.

A central assumption from an ecological point of view is that humans are “informavores”, that ingest information^47^. According to this interpretation, which does not assume the existence of internal models, the postural control system actively searches for information by purposefully generating sensory feedback to estimate the equilibrium state of the body. This “active” sensation and perception has been defined by Bajcsy^48^ as an intelligent sensory data acquisition process. In the domain of postural control, Riccio and Stoffregen^49^ demonstrated that active perception of body orientation is grounded on the perception of corrective actions required to keep a chosen tilted or listing posture. They suggested that a trade-off between less effortful postural corrections when nearer to the equilibrium point and more reliable interpretation of the forces acting on the body with greater deviation from the equilibrium point governs postural control^49^. Similarly, Riley et al.^16^ contrasted body sway dynamics during normal upright standing and forward leaning and concluded that the short-term dynamics of normal standing consists of a greater amount of exploratory behaviour due to the greater distance to the limits of stability. An extension of the ecological point of view was proposed by Stoffregen and co-workers^14^. They assumed that postural control takes part as a component of a purposeful perception-action coupling, so that body sway is actively modulated to assist in any suprapostural tasks requiring oculomotor or tactile precision^50–52^.

Furthermore, some modeling approaches sucessfully simulate patterns of human behaviour, e.g. sway patterns, by a pure feedback control model^53^. However, anticipation of situations and movements in the near future are important to reduce time delay^54,55^ and to create smoother trajectories^56^. Moreover, the exploratory behaviour described in previous studies^15,17^, has also been discussed in relation to the feed-forward control of the two-fold control mechanisms underlying postural control^16^, which was first proposed as open-loop control time-periods by Collins and De Luca^35^ by performing a stabilogram diffusion analysis on centre of pressure (CoP) measurements.

Since knowledge of the size and weight of the own body and its segments, and especially about its current inertia and postural configuration in space in a given situation are important regarding the control systems’ approach^30,31^ when moving, body schema is discussed as a factor that influences motor control^31^ in general, and postural control more specifically^33,34^. Moreover, body schema and body image have been discussed to interact with each other in a co-constructive manner and reshape one another^30^.

To better understand inter-individual differences in body sway control, the aim of this study is to investigate the relationship between body representations and postural control and the influence of a participant’s gender on the role of associated factors. We expect body image and body schema to be related to postural control in addition to the commonly reported influencing factors, such as sensory condition, age, gender and anthropometry. Furthermore, gender differences are expected to alter the role of anthropometry and body representations on body sway control.

This study is the first directly testing the association of high-order body representations with postural control. We discuss how high-level representations of the self, both body image and body schema, may be involved in predictive human body sway control and how this relationship may be moderated by gender and biological sex. In order to investigate the relationship of these factors we conduct a hierarchical regression analysis of body sway parameters with measures of known influencing factors and aspects of body representations.

## Results

### Relationship between body representation and postural control

#### Body sway variability

**Figure 1:**
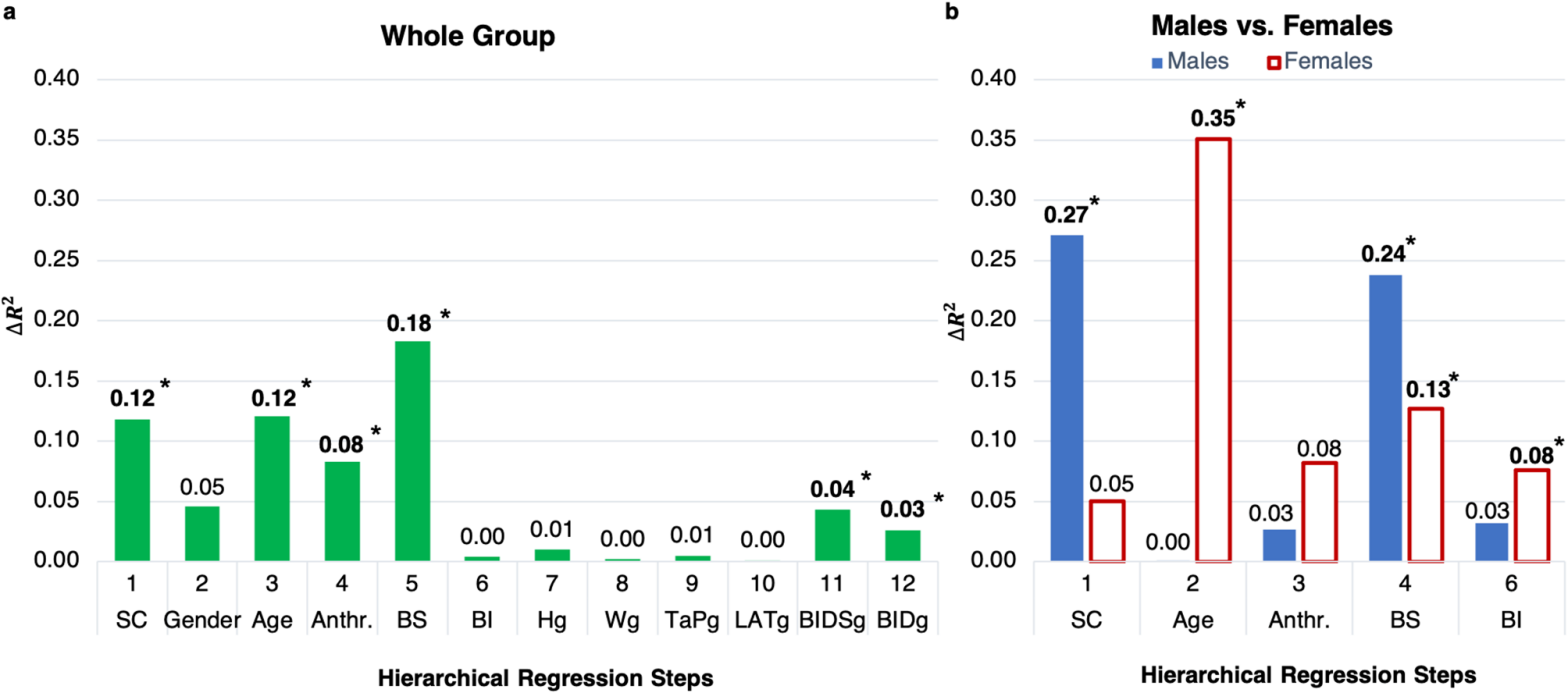
Regression results (entry method) for whole group (**a**) and for gender groups (**b**). $\Delta$ R$^2$ for each hierarchical regression step. SC = sensory condition (eyes closed (EC); neck extended, eyes closed (NE-EC)); Gender (male; female); Anthr. = anthropometry (height (H); weight (W)); BS = body schema (${TaP}_{\mathrm{v}}$; ${LAT}_{\mathrm{a}}$); BI = body image ($\hbox {BIDS}_{\mathrm{abs}}$; $\hbox {BID}_{\mathrm{abs}}$); gender-interactions: Hg, Wg, TaPg, LATg, BIDSg, BIDg. Bold with star indicates significance ($P\le 0.05$).

For all 36 subjects (19 females), body schema measures (overt task: inverted *Taking-a-Posture task* variability (${TaP}_{\mathrm{v}}$), covert task: *Laterality* accuracy (${LAT}_{\mathrm{a}}$)) significantly increase the goodness of fit ($R^2$) of the hierarchical multiple linear regression model ($\Delta$F(2,64) = 13.11, $P\le$0.01), in addition to commonly influencing factors, such as sensory condition, sex/gender, age and anthropometry (Fig. 1a). Subjects’ gender was equal to their sex. Therefore, we refer to both their sex and gender.

Figure 1 represents the results for the individual regression steps:

1. Sensory condition: EC (eyes closed); NE-EC (neck extended, eyes closed)
2. Gender: male; female
3. Age
4. Anthropometry: body height; body weight
5. Body schema (BS): overt (${TaP}_{\mathrm{v}}$); covert (${LAT}_{\mathrm{a}}$)
6. Body image (BI): absolute body image dissatisfaction ($\hbox {BIDS}_{\mathrm{abs}}$); absolute body image distortion ($\hbox {BID}_{\mathrm{abs}}$)
7. Gender-interactions: gender-height ($\hbox {H}_{\mathrm{gen}}$); gender-weight ($\hbox {W}_{\mathrm{gen}}$); gender-${TaP}_{\mathrm{v}}$ ($\hbox {TaP}_{\mathrm{gen}}$); gender-${LAT}_{\mathrm{a}}$ ($\hbox {LAT}_{\mathrm{gen}}$); gender-$\hbox {BIDS}_{\mathrm{abs}}$ ($\hbox {BIDS}_{\mathrm{gen}}$); gender-$\hbox {BID}_{\mathrm{abs}}$ ($\hbox {BID}_{\mathrm{gen}}$)

**Figure 2:**
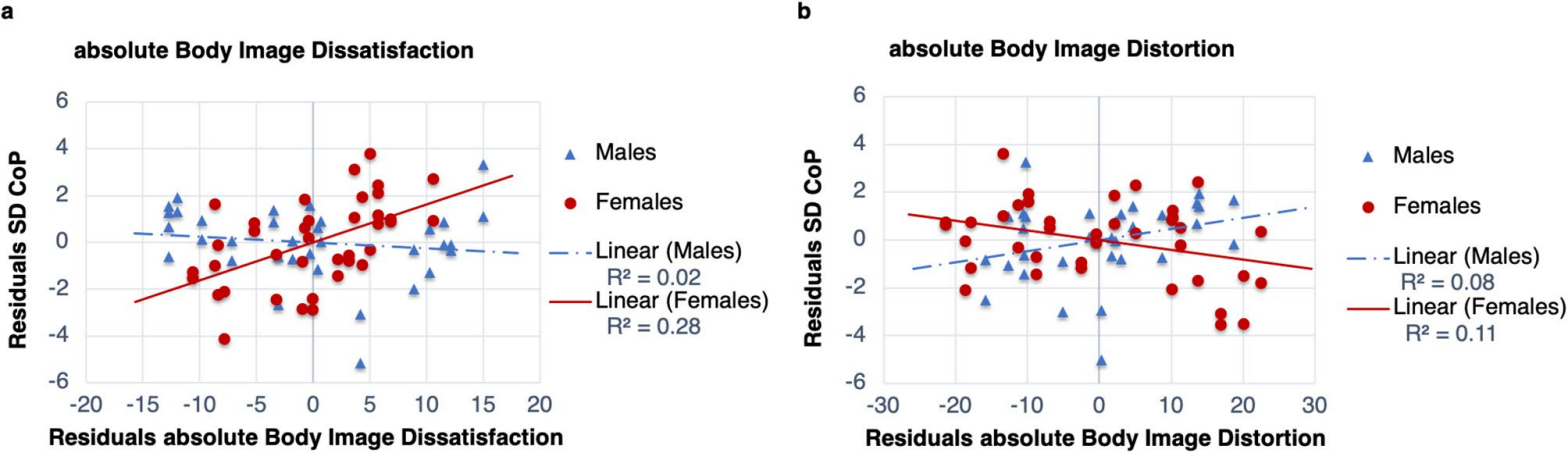
Gender-interactions: partial regression plots of absolute body image dissatisfaction (**a**) and absolute body image distortion (**b**).

**Figure 3:**
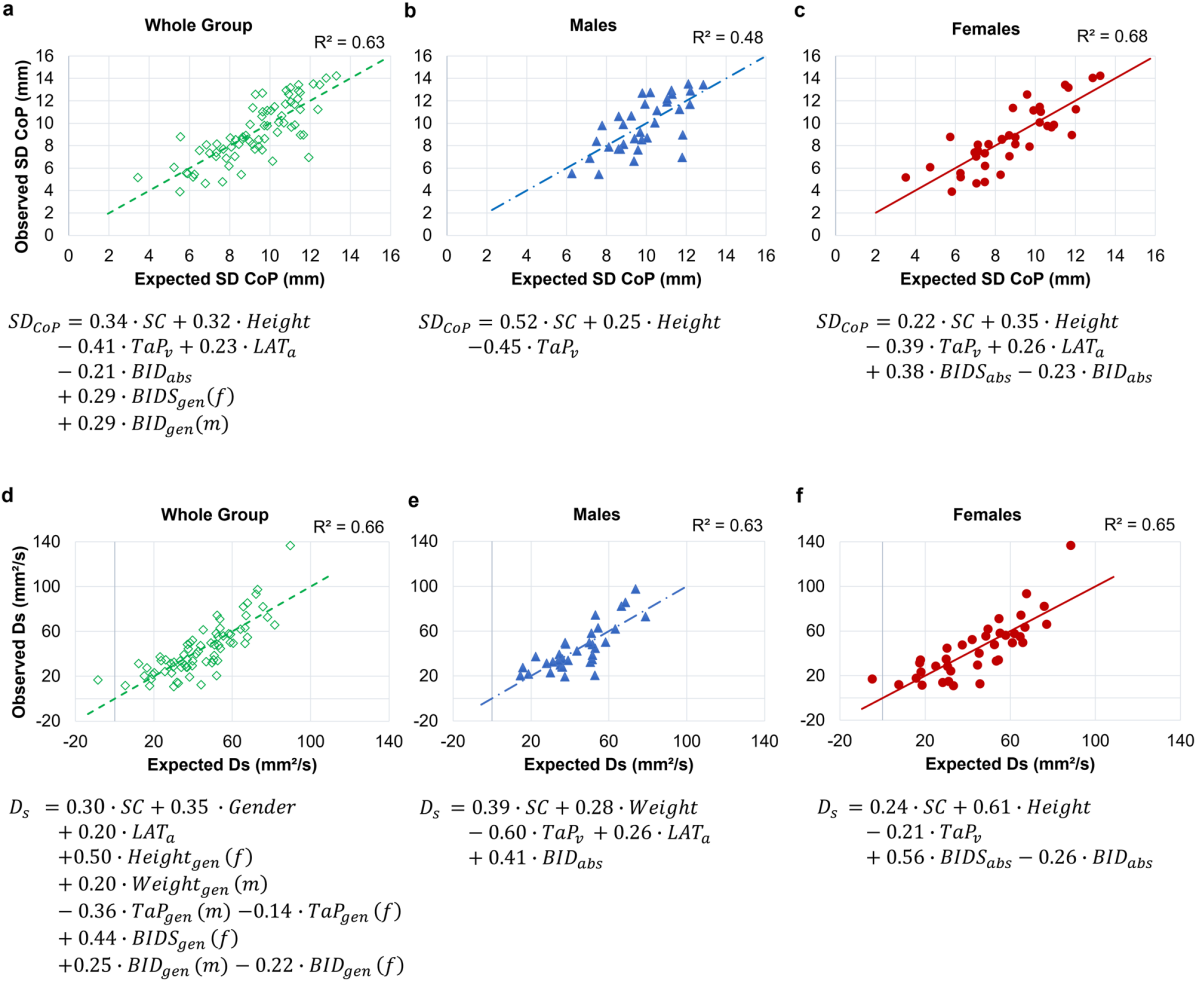
Best regression plots with minimal number of predictors. Sway variability (SD CoP): upper row (**a**–**c**); Short-term stochastic activity ($\hbox {D}_{\mathrm{s}}$): lower row (**d**–**f**); whole group (**a**, **d**): gender-interactions ($_{\mathrm{gen}}$): for males (m), e.g. $\hbox {BIDS}_{\mathrm{gen}} = 0$; for females (f), e.g. $\hbox {BID} _{\mathrm{gen}} = 0$; males (**b**, **e**); females (**c**, **f**).

Most of the common influencing factors significantly increase the goodness of fit: sensory condition ($\Delta$F(1,70) = 9.37, $P\le 0.01$), age ($\Delta$F(1,68) = 11.57, $P\le 0.01$), and anthropometry ($\Delta$F(2,66) = 4.34, $P = 0.02$). On the other hand, indirect body image measures ($\hbox {BIDS}_{\mathrm{abs}}$, $\hbox {BID}_{\mathrm{abs}}$) do not significantly increase $R^2$ ($\Delta$F(2,62) = 0.27, $P = 0.77$). While gender itself does not significantly increase the goodness of fit ($\Delta$F(1,69) = 3.83, $P = 0.06$), gender-interactions (moderation of the influence of a variable on body sway by gender) with $\hbox {BIDS}_{\mathrm{abs}}$, $\hbox {BID}_{\mathrm{abs}}$ significantly increase the goodness of fit ($\Delta$F(1,57) = 6.32, $P = 0.02$; $\Delta$F(1,56) = 4.10, $P = 0.05$, respectively) (Figs. 1b and 2). The best model with minimal number of explanatory variables (explanans/explanantia) (backward model) reveals a goodness of fit of 62.7% (F(7,64) = 15.36, $P\le 0.01$) (Fig. 2). A decreased body sway variability is associated with a better overt *TaP* task performance (less variable = higher inverted ${TaP}_{\mathrm{v}}$) (${t}(64) = -4.51$, $P \le 0.01$, $pr^2$ = 0.24, $f^2$ = 0.32, *SP* = 0.90), and with a more distorted body image (${t}(64) = -2.03$, *P* = 0.05, $pr^2$ = 0.06, $f^2$ = 0.06, *SP* = 0.31). On the other hand, an increased body sway variability is associated with a more difficult sensory condition (SC) (NE-EC) (${t}(64) = 4.50$, $P\le 0.01$, $pr^2$ = 0.24, $f^2$ = 0.32, *SP* = 0.90), with an increased height, (${t}(64) = 3.91$, $P\le 0.01$, $pr^2$ = 0.19, $f^2$ = 0.23, *SP* = 0.80), and with a better covert task (*LAT*) performance (${t}(64) = 2.71$, $P\le 0.01$, $pr^2$ = 0.10, $f^2$ = 0.11, *SP* = 0.49). Finally, the interactions of gender with body image dissatisfaction (females: ${t}(64) = 3.72$, $P\le 0.01$, $pr^2$ = 0.18, $f^2$ = 0.22, *SP* = 0.77) and body image distortion (males: ${t}(64) = 2.80$, $P\le 0.01$, $pr^2$ = 0.11, $f^2$ = 0.12, *SP* = 0.53) contribute significantly to the model. In summary, the overt *TaP* task performance results to be the best explanans, followed by sensory condition, height, the gender-$\hbox {BIDS}_{\mathrm{abs}}$ interaction, the gender-$\hbox {BID}_{\mathrm{abs}}$ interaction, the covert *LAT* task performance and the $\hbox {BID}_{\mathrm{abs}}$ (see standardised regression weights in Fig. 3a).

#### Short-term stochastic activity

The best regression model (backward model) for the short-term stochastic activity of body sway dynamics, which is represented by the short-term diffusion coefficient ${D}_{s}$, reveals a goodness of fit of 65.8% (F(10,61) = 11.75, $P\le 0.01$) (Fig. 2). Due to homoscedascity, parameter estimates are reported as adjusted for robust standard errors. A greater short-term stochastic activity is associated with a more difficult sensory condition (NE-EC) (${t}_{{a}}(61) = 3.73$, ${P}_{{a}}\le 0.01$, ${p}{\eta } ^{2} = 0.19$, *oSP* = 0.96), with females compared to males, (${t}_{{a}}(61) = 3.33$, ${P}_{{a}}\le 0.01$, ${p}{\eta } ^{2} = 0.15$, *oSP* = 0.91), and with a better covert LAT task performance (*t*_a_(61) = 2.73, *P*_a_ ≤ 0.01, *pη*^2^ = 0.11, *oSP* = 0.77). Finally, the interactions of gender with the overt *TaP* task performance (males: ${t}_{{a}}(61) = -3.39$, ${P}_{{a}}\le 0.01$, ${p}\eta ^2 = 0.16$, *oSP* = 0.92); females: ${t}_{{a}}(61) = -2.09$, ${P}_{{a}} = 0.04$, ${p}\eta ^2 = 0.07$, *oSP* = 0.54), with height (females: ${t}_{{a}}(61) = 5.92$, ${P}_{{a}}\le 0.01$, ${p}\eta ^2 = 0.37$, *oSP* = 1.00), with weight (males: ${t}_{{a}}(61) = 3.95$, ${P}_{{a}}\le 0.01$, *p* $\eta$ $^2$ = 0.20, *oSP* = 0.97), and with body image dissatisfaction (females: ${t}_{{a}}(61) = 3.79$, ${P}_{{a}}\le 0.01$, ${p}\eta ^2 = 0.19$, *oSP* = 0.96) as well as with the body image distortion (males: ${t}_{{a}}(61) = 4.06$, ${P}_{{a}}\le 0.01$, ${p}\eta ^2 = 0.21$, *oSP* = 0.98; females: *t*_a_(61) = −2.22, *P*_a_ = 0.03, *p*η^2^ = 0.08, *oSP* = 0.59) contribute significantly to the model. Standardised regression coefficients calculated based on the unstandardised regression coefficient B corrected by the robust standard error are shown in Fig. 3d).

### Gender effect on the relationship between influencing factors and postural control

#### Body sway variability

As indicated by the previously stated gender-interactions, we observe sex/gender differences when dividing the group into males and females (Figs. 1b and 2). In males (N = 17, age: 20–32), sensory condition ($\Delta$*F*(1,32) = 11.87, *P* $\le$ 0.01) and body schema ($\Delta$*F*(2,27) = 6.95, *P* $\le$ 0.01) significantly increase the goodness of fit (Fig. 1b). The best model of the CoP variability with minimal number of explanatory variables (backward model) reveals a goodness of fit of 48.4% (F(3,30) = 9.38, *P* $\le$ 0.01) (Fig. 2). A decreased body sway variability is associated with a better *TaP* task performance (${t}(30) = -3.32$, *P* $\le$ 0.01, *pr* $^2$ = 0.27, *f* $^2$ = 0.37, *SP* = 0.65). On the other hand, an increased body sway variability is associated with a more difficult sensory condition (${t}(30) = 3.97$, *P* $\le$ 0.01, *pr* $^2$ = 0.35, *f* $^2$ = 0.54, *SP* = 0.80) and with an increased height (*t*(30) = 1.87, *P* = 0.07, *pr* $^2$ = 0.10, *f* $^2$ = 0.11, *SP* = 0.25). In males, sensory condition reveals to be the best explanans, followed by the overt (*TaP*) task performance, and finally by height (Fig. 3b).

On the other hand, females (N = 19, age: 18-30) have demonstrated more factors to contribute substantially to the explanation of inter-and intrapersonal variability; age ($\Delta$F(1,35) = 20.51, *P* $\le$ 0.01) body schema ($\Delta$F(2,31) = 5.08, *P* $\le$ 0.01) and body image measures ($\Delta$F(2,29) = 3.50, *P* = 0.04) significantly increase the goodness of fit (Fig. 1b). The best model with minimal number of explanatory variables (backward model) reveals a goodness of fit of 68.0% (F(6,31) = 10.98, *P* $\le$ 0.01) (Fig. 2). A decreased body sway variability is associated with a better overt (*TaP*) task performance (${t}(31) = -3.56$, *P* $\le$ 0.01, *pr* $^2$ = 0.29, *f* $^2$ = 0.41, *SP* = 0.73), and with a greater body image distortion (${t}(31) = -1.99$, *P* = 0.06, *pr* $^2$ = 0.11, *f* $^2$ = 0.12, *SP* = 0.29). On the other hand, an increased body sway variability is associated with a more difficult sensory condition (*t*(31) = 2.21, *P* = 0.04, *pr* $^2$ = 0.14, *f* $^2$ = 0.16, *SP* = 0.37), with an increased height (*t*(31) = 3.04, *P* $\le$ 0.01, *pr* $^2$ = 0.23, *f*$^2$ = 0.30, *SP* = 0.59), with a better covert (*LAT*) task performance (*t*(31) = 2.37, *P* = 0.02, *pr* $^2$ = 0.15, *f* $^2$ = 0.18, *SP* = 0.39), and with an increased $\hbox {BIDS}_{\mathrm{abs}}$ (*t*(31) = 3.46, *P* $\le$ 0.01, *pr* $^2$ = 0.28, *f* $^2$ = 0.39, *SP* = 0.70) (Fig. 3c). For females the overt (*TaP*) task performance reveals to be the best explanans, followed by body image dissatisfaction, height, the covert (*LAT*) task performance, body image distortion, and finally by the sensory condition.

#### Short-term stochastic activity

When looking at the best model (backward model) of the diffusion coefficient in males, it reveals a goodness of fit of 63.1% (F(5,28) = 9.57, *P* $\le$ 0.01) (Fig. 2). A decreased short-term stochastic activity is associated with a better *TaP* task performance (${t}(28) = -3.94$, *P* $\le$ 0.01, *pr* $^2$ = 0.36, *f* $^2$ = 0.56, *SP* = 0.80). On the other hand, an increased short-term stochastic activity is associated with a more difficult sensory condition (*t*(28) = 3.40, *P* $\le$ 0.01, *pr* $^2$ = 0.29, *f* $^2$ = 0.41, *SP* = 0.67), with an increased weight (*t*(28) = 2.31, *P* = 0.03, *pr* $^2$ = 0.16, *f* $^2$ = 0.19, *SP* = 0.38), with a better covert *LAT* task performance (*t*(28) = 1.92, *P* = 0.07, *pr* $^2$ = 0.12, *f* $^2$ = 0.14, *SP* = 0.29) and with an increased $\hbox {BID}_{\mathrm{abs}}$ (*t*(28) = 2.87, *P* ≤ 0.01, *pr* $^2$ = 0.23, *f* $^2$ = 0.30, *SP* = 0.54). For the short-term stochastic activity the overt *TaP* task performance reveals to be the best explanans, followed by the body image distortion, the sensory condition, weight, and finally by the covert *LAT* task performance (Fig. 3e).

In females, the minimal model of short-term stochastic activity reveals a goodness of fit of 65.1% (F(5,32) = 11.96, *P* $\le$ 0.01) (Fig. 2). A decreased short-term stochastic activity is associated with an increased overt (*TaP*) task performance (${t}(32) = -2.48$, *P* = 0.02, *p* $\eta$ $^2$ = 0.16, *oSP* = 0.67), and with an increased body image distortion (${t}(32) = -2.20$, *P* = 0.04, *p* $\eta$ $^2$ = 0.13, *oSP* = 0.57). On the other hand, an increased short-term stochastic activity is associated with a more difficult sensory condition (*t*(32) = 2.14, *P* = 0.04, *p* $\eta$ $^2$ = 0.13, *oSP* = 0.55), with an increased height (*t*(32) = 6.21, *P* $\le$ 0.01, *p* $\eta$ $^2$ = 0.55, *oSP* = 1.00), and with an increased $\hbox {BIDS}_{\mathrm{abs}}$ (*t*(32) = 3.97, *P* $\le$ 0.01, *p* $\eta$ $^2$ = 0.33, *oSP* = 0.97) (Fig. 3f). For females, this time, height reveals to be the best explanans, followed by body image dissatisfaction, body image distortion, by the sensory condition and finally by the overt *TaP* task performance.

## Discussion

This study is the first directly testing the association of body representations, such as body schema and body image, with postural control. For the interpretations it has to be taken in mind that high-order body representations cannot be directly observed but only inferred by indirect measures.

Our study revealed that body schema measures are associated with human postural control in addition to previously reported influencing factors, such as sensory condition, sex/gender, age and anthropometry. Further, the regression models demonstrate that sex/gender moderates the role of high-order body representations in postural control; body image dissatisfaction being related to body sway variability and open-loop control only in females.

Interestingly, when looking at indirect body image measures, we observed that it is rather body image dissatisfaction than body image distortion which seems to play a role in postural control in females. The work of Forghieri et al.^27^ showed a higher sway increase in female patients with eating disorders compared to controls, when they saw their mirror image or ideal model image during quiet standing. Moreover, patients also showed a greater body dissatisfaction^27^. This points to a similar relationship between body image and postural control. However, Forghieri et al.^27^ did not see differences in body sway in patients and controls during eyes closed condition, which was always assessed before the body image trials^27^. In contrast to their study, in our study, the balance and overt *TaP* tasks were always performed after the body image task. Thus, it might be that the conscious access of individuals’ body image and dissatisfaction with their body remained active during the balance task in females. Moreover, Williams et al.^57^ have reviewed the relationship between sensorimotor control and emotions. They proposed that emotional self-awareness can be attributed to interoceptive awareness and that emotions affect internal models of sensorimotor-goal relationships^57^. Therefore, the interoceptive awareness as part of attention might influence our body image via emotions and consequently the body and sensory dynamics of the internal model potentially involved in postural control. In analogy to the affective-signaling theory by Dignath and colleagues^58^ in the field of conflict monitoring and cognitive control, which assumes negative affect to be linked to adaptations in cognitive control, similar mechanisms may occur also in postural control.

Even though, body image dissatisfaction resulted to be stronger related to postural control in females (see standardised regression weights in Fig. 3), also body image distortion contributed substantially to the regression model in both females and males; for latter only for short-term stochastic activity. Thereby, an increased absolute body image distortion coincides with a reduced body sway variability and short-term exploratory behaviour in females. This might be explained by an overshooting of torque in relation to the torque needed^59^, due to controlling for an illusionary increased body mass (overestimated body mass in the internal model). However, effect size was small to medium and power was low.

While on the other hand, even though obese individuals show an increased torque compared to non-obese, they might underestimate the torque needed to stabilise their body (undershooting of torque)^60^. The observed undershooting of torque in obese patients might also be attributed to a misperception and thus underestimation of their weight status^61^. Consequently, the increased stability due to especially a sudden weight loss (due to surgery) in obesity^62^, might thus be related not only to the reduced weight itself, but also to a reduced underestimation of body size, and consequently to a reduced undershooting of torque. Teasdale et al.^62^ also related increased stability with weight loss in obese males to an increased capacity of the anticipatory system and of the multisensory integration. They argued this to be possibly associated with a better detection of changes with respect to verticality due to a smaller contact area^62^. Our results observed in the regression of the diffusion coefficient representing the level of short-term exploratory behaviour may support this, as males showed lower body image distortion to be associated with less exploratory behaviour. This indicates less short-term exploratory behaviour and thus potentially a better feed-forward model with a less distorted body image in males. However, there was no substantial relationship observed for sway variability and also power for explaining short-term exploratory behaviour was low. Moreover, the lower relative body image distortion and higher proportion of individuals underestimating their own body size in males compared to women (29.4% vs. 10.5%, respectively) may be a reason for the opposite relationship observed for temporal sway parameters in males and in females. Therefore, it might be possible that also or especially in males body image distortion plays a role in postural control, when it is increased (e.g. increased underestimation).

Gender differences in body image might be attributed to the substantially higher overestimation of body size (see relative BID in Table 2) and the slightly higher drive for thinness (see relative BIDS in Table 2) in females. While the wish to be thinner is attributed to negative emotions^27^, the wish to be wider or more muscular, which we observed to be higher in males than in females (see DMS in Table 1), might not be affected that much by negative feelings. As previously discussed, negative affect may play a role in adaptations of control mechanisms. Thus, it remains unclear if eventually also in males body image dissatisfaction might be related to postural control when this is attached with more negative emotions (e.g. increased drive for thinness).

**Table 1:** Subject characteristics and group comparisons (t-tests and Mann–Whitney-U tests) for age, anthropometrics, psychometrics, and body-related experiences.

Variables	Whole group (n = 36) mean ± SD	Males (n = 17) mean ± SD	Females (n = 19) mean ± SD	Significance (gender comparison)
Age (years)	26.39 ± 3.14	27.24 ± 2.69	25.63 ± 3.36	0.08
**Anthropometrics**				
Height (m)	1.74± 0.07	1.77 ± 0.04	1.72 ± 0.07	≤ **0.01**
Weight (kg)	66.16 ± 9.30	72.46 ± 6.72	60.53 ± 7.53	≤ **0.01**
BMI (kg/m$^2$)	21.79 ± 2.53	23.17 ± 2.16	20.57 ± 2.19	≤ **0.01**
Leg length (m)	0.91 ± 0.05	0.94 ± 0.03	0.89 ± 0.05	≤ **0.01**
HC (cm)	0.96 ± 0.05	0.95 ± 0.05	0.96 ± 0.06	0.62
WC (m)	0.75 ± 0.08	0.80 ± 0.07	0.71 ± 0.06	≤ **0.01**
ShC (m)	1.03 ± 0.07	1.10 ± 0.03	0.97 ± 0.04	≤ **0.01**
HWR	1.28 ± 0.12	1.19 ± 0.07	1.37 ± 0.08	≤ **0.01**
SWR	1.38 ± 0.10	1.38 ± 0.12	1.38 ± 0.09	0.70
**Psychometrics**				
RSES	23.97 ± 3.90	24.00 ± 4.65	23.95 ± 3.15	0.59
PACS	13.92 ± 2.03	13.94 ± 1.79	13.89 ± 2.25	0.58
DMS	31.94 ± 9.19	35.53 ± 7.99	28.74 ± 9.10	≤ **0.01**
**Body-related experience**				
FreqSports	3.31 ± 1.50	3.41 ± 1.52	3.21 ± 1.49	0.55
FreqMirror	2.00 ± 1.30	1.35 ± 1.20	2.58 ± 1.15	≤ **0.01**

*HC* hip circumference, *WC* waist circumference, *ShC* shoulder circumference, *HWR* hip waist ratio, *SWR* shoulder waist ratio, *RSES* Rosenberg Self Esteem Scale, *PACS* Physical Appearance Comparison Scale, *DMS* Drive for Muscularity Scale, *FreqSports* frequency of sports per week, *FreqMirror* frequency of seeing own body in the mirror, Frequency: 0 = never; 1 = 1x; 2 = 2x; 3 = 3x; 4 = 4x; 5 = min. 5x.

Significant values are in bold.

Besides gender differences in body image, we also observed differences between males and females in the relevance of the sensory condition and age in explaining intra- and inter-individual variability in body sway. While sensory condition had a stronger relationship with both sway variability and short-term exploratory behaviour in males than in females (see Figs. 1 and 3), age affected body sway substantially only in females (see Fig. 1). The difference in sensory condition can be also observed in Table 2, which shows a substantially higher body sway variability in the neck extended condition in males compared to females. Sex differences in vestibular functioning were also proposed by Nolan and colleagues^6^ to possibly explain differences in 9-10 year old boys and girls during quiet standing with eyes closed. The lower change in sway variability in females compared to males by extending the neck in the current study suggests that females either rely more on proprioceptive and tactile information or are more sensitive to vestibular disruption. A higher sensitivity to vestibular changes has been also reported in patients with vestibular migraine, who are susceptible to motion sickness^63^. Moreover, the incidence for motion sickness has been predominantly observed in females^5^. Thus, females in the current study might have adapted quicker to changes in the neck extended condition than males due to a higher sensitivity, or they might have downgraded the vestibular system due to relying less on vestibular information which is the more plausible explanation, since females did not change the control strategy. Regarding the ecological theory of postural instability and motion sickness^64^ a generally greater reliance on proprioceptive and tactile information may dynamically constrain postural control during standing on a stable surface, like in our study, while eventually inducing a greater postural instability and thus increase susceptibility to motion sickness when standing on a moving surface.

**Table 2:** Subject characteristics and group comparisons (t-tests and Mann–Whitney-U tests) for body representations (body image, body schema) and body sway.

Variables	Whole group (n = 36) mean ± SD	Males (n = 17) mean ± SD	Females (n = 19) mean ± SD	Significance (gender comparison)
**Body image**				
BID relative (%)	15.44 ± 19.30	9.10 ± 18.49	21.11 ± 18.44	≤ **0.01**
BIDS relative (%)	− 5.07 ± 13.69	− 1.38 ± 16.64	− 8.37 ± 9.43	0.16
BID absolute (%)	20.19 ± 14.18	16.45 ± 12.19	23.53 ± 15.14	0.06
BIDS absolute (%)	11.98 ± 8.25	13.29 ± 9.85	10.81 ± 6.41	0.27
**Body schema**				
*Taking-a-posture* task				
*TaP* accuracy	− 12.91 ± 2.75	− 12.91 ± 2.18	− 12.92 ± 3.20	0.60
*TaP* variability	− 1.31 ± 0.41	− 1.39 ± 0.42	− 1.24 ± 0.40	0.09
*Laterality* task				
*FLAT* accuracy(%)	57.67 ± 37.00	65.67 ± 36.01	50.51 ± 36.87	0.17
*FLAT* latency(s)	1.76 ± 0.94	1.68 ± 0.77	1.83 ± 1.07	0.60
*HLAT* accuracy(%)	84.90 ± 27.31	82.48 ± 31.62	87.06 ± 23.00	0.46
*HLAT* latency(s)	1.21 ± 0.46	1.15 ± 0.45	1.26 ± 0.48	0.23
*LAT* accuracy(%)	71.28 ± 25.86	74.07 ± 28.90	68.79 ± 22.91	0.22
*LAT* latency (s)	1.48 ± 0.65	1.42 ± 0.54	1.54 ± 0.74	0.51
**Body sway**				
*SD CoP (mm)*	9.27 ± 2.62	9.86 ± 2.37	8.74 ± 2.75	0.07
In EC	8.38 ± 2.29	8.65 ± 1.91	8.13 ± 2.61	0.51
In NE-EC	10.16 ± 2.65	11.08 ± 2.18	9.35 ± 2.82	**0.05**
${D}_{{s}}$ (mm$^2$/s)	43.97 ± 23.45	44.01 ± 20.11	43.94 ± 26.36	0.79
In EC	37.03 ± 16.33	36.28 ± 12.83	37.70 ± 19.26	0.80
In NE-EC	50.91 ± 27.38	51.74 ± 23.29	50.17 ± 31.21	0.87
${H}_{{s}}$ (mm$^2$/s)	0.83 ± 0.03	0.83 ± 0.03	0.83 ± 0.03	0.59
In EC	0.83 ± 0.03	0.83 ± 0.04	0.83 ± 0.03	0.95
In NE-EC	0.83 ± 0.03	0.83 ± 0.03	0.82 ± 0.04	0.40
$${TP}_{t} (s) $$	1.15 ± 0.27	1.14 ± 0.27	1.16 ± 0.28	0.70
In EC	1.12 ± 0.29	1.15 ± 0.31	1.10 ± 0.27	0.66
In NE-EC	1.18 ± 0.26	1.13 ± 0.23	1.22 ± 0.27	0.28

*BID* body image distortion, *BIDS* body image dissatisfaction, *TaP* Taking-a-posture task, *LAT* laterality task, *FLAT* foot laterality task, *HLAT* hand laterality task, *SD CoP* standard deviation of centre of pressure, *D*_*s*_ short-term diffusion coefficient, *H*_*s*_ short-term Hurst exponent, *TP*_*t*_ transition time point, *EC* eyes closed, *NE-EC* neck extended, eyes closed.

Significant values are in bold.

Furthermore, sex differences in the influence of age (see Fig. 1) might be due to a slightly higher variance in females than in males (see Table 1), as well as due to slightly lower age in females (18–30 years) than in males (20–32 years). This might influence the relationship due to possible growth in the younger adults or other developmental factors. Moreover, it is in line with previous results of age effect^2^, which showed steepest sway reduction in the age of 7–25 years and then a plateau until the age of 55. However, the age effect disappeared in the final model (see Fig. 3), when also the indirect body schema and body image measures were considered. This indicates a possible mediation effect on the influence of age on body sway via body schema and body image. This is supported by the influence of increased movement experience on body schema^32^, as well as by a reduced body image dissatisfaction with increased age in females^65^. In this context, we can also observe a substantial moderate positive correlation of age with the overt body schema measure, and moderate negative correlations with the covert body schema measure and absolute body image dissatisfaction in females (see additional correlation results in Supplementary Table S5). Additional correlation analysis for males and the whole group can be found in the Supplementary Materials (Supplementary Tables S3 and S4).

The second type of hypothetical high-order body representation investigated in this study, the body schema, has been previously discussed as a self-reference which is fed into different control strategies as a reference for motor control^31^ providing the state estimate^34^. In postural control, state estimation errors have been proposed to cause the slow dynamics, which represents the majority of body sway and is located within the feedback loop^37^. The task performance in the overt *Taking-a-Posture* is associated with human body sway and postural control in both males and females. On the other hand, even though the covert *Laterality* task contributed substantially in the regression model of sway variability in the whole group without a substantial gender interaction (see Fig. 3a), it did not contribute substantially in males when computed the regression models of sway variability separately for gender (see Fig. 3b). This might be due to a smaller number of participants in males than in females. On the other hand, for the short-term diffusion coefficient the covert task performance contributed only to the model in men. However, effect sizes were again only small to medium and power was. The contribution of both task performances, we observed, is in line with Barrato et al.^36^, who stated postural control to consist of two components: (1) multisensory integration for estimating the on-line centre of mass (CoM) position (internal model including feedback), and (2) prediction of the CoM displacement based on an internal model to compensate intrinsic sensory delays (feed-forward without feedback).

The higher contribution of the overt body schema task for explaining postural control (see standardised regression weights in Fig. 3a, c) indicates the importance of continuously updating the internal model by sensory feedback, such as from proprioceptive and tactile sensors^66^, for maintaining postural control. Further, the influence of a potential body schema on body sway might be moderated by coordination- and balance-related sports types, since dancers show better performance in proprioceptive tasks^43^, an increased anticipatory control in dynamic tasks^42^ and automated feedback-control in static tasks^67^, due to higher sensitivity to small changes. Moreover, proprioceptive awareness^44^ and self-awareness^42^ have been reported to be higher in e.g. dancers, which would increase the sensory sensitivity to smaller changes and increase their fast dynamics^67^. Thus, dancers for example might not only have a very accurate internal model^42^, but they may also quickly adapt to very small deviations^67^.

However, the positive relationship of the covert *Laterality* accuracy parameter and body sway in the current study resulted in the opposite than expected; instead of a more accurate *Laterality* task performance relating to a lower body sway, we observed higher body sway variability and short-term exploratory behaviour. A typical observation made in healthy adults and investigated in the context of body schema is the effect of biomechanical constraints on the accuracy and response time in the *Laterality* task^68,69^ as well as the correlation of the response time and the movement time when performing a real hand rotation to match the position of a shown hand^68^. Thus, it might be possible that even though subjects were instructed to imagine to observe their own limbs, instead of using a motor imagery strategy they might have used an alternative strategy. For example Creem-Regehr et al.^70^ have reported a higher accuracy in a viewer (perspective transformation) strategy compared to a hand rotation strategy. One explanation, thus could be, that for healthy individuals, like in the current study, a higher accuracy instead of indicating a better body schema might rather indicate e.g. that they might have used an alternative strategy in the biomechanically implausible rotations of the limb instead of a motor imagery strategy^69^. On the other hand, individuals born with only one hand (congenital one-handers), who are expected to have only a motor representation for the existing hand, have shown a lower accuracy in the *Laterality* task^71^ together with a longer response latency compared to controls and individuals with acquired hand loss. Maimon-Mor et al.^71^ have concluded the current motor control to be a driver in the hand laterality performance. Moreover, Schwoebel et al.^46^ stated that the task performance in both motor imagery and real movements might partly rely on body schema. On the other hand an increased short-term stochastic activity related to the covert task performance may indicate more exploratory movements^15–17^ to gain more information for improving task performance in both postural control and motor imagery.

Pitron et al.^30^ have proposed a serial model in which body schema and body image reshape each other. Regarding their hypothesis, body schema is first developed by prior motor experiences and multi-sensory integration. Subsequently, body schema develops and shapes body image together with again multisensory integration as well as other priors, such as cultural and social expectations, which in turn reshape body schema^30^. Consequently, body image and the covert aspect of body schema (without movement-induced feedback) might influence the internally simulated body and sensor dynamics. Based on an internal model, a state estimate is sent to the controllers, as proposed by Kuo^34^ and Morasso et al.^31^. The control strategy used and the weighting of the feed-forward and feedback control might be influenced by the movement strategy and cognitive processing, as factors reported to influence postural control^25^. The motor commands consequently cause body dynamics^34^, which can be restricted by biomechanical constraints^25,72^ and gravity^31^. The body dynamics causes sensory dynamics, based on which the sensory output will be compared with the predicted sensory output. Furthermore, based on the observations in expert dancers , for example, it is known that also proprioceptive awareness, as part of body schema, can reach an expert level and that dancers rely more on proprioceptive information than non-dancers^42–44,67^. This is also supported by Gallagher^33^ and Fabre et al.^42^, who stated that body schema and proprioceptive awareness share same information, and that a higher proprioceptive awareness improves the internal model, respectively. Thus, we suppose that body schema and the sensory proprioceptive awareness influence the sensory noise in overt actions. Consequently, this also might affect the estimator gain, which causes an estimator correction of the internal model^34^.

Besides these interpretations also other interpretations are possible, which are not necessarily based on internal representations. Thus, for example, it could be that differences between males and females are due to different *perception-action couplings*^50–52^. However, future studies would be needed to further investigate and discuss the role of body representations, feed-forward and feedback control in postural control.

### Limitations and future works

The sample population in the current study consisted of healthy young adults with most of them performing regular sports, at least once per week. Thus, caution has to be taken when generalising the results to the average population. Since this was a first proof-of-concept study about the correlation of high-order body representations and postural control, we can not conclude about a cause-effect relationship and results have to be interpreted with caution. We have not included the type of sports for recruiting and only created groups for different sport types post-hoc based on the responses of the participants (see Supplementary Table S1). In addition to a possible moderation of the effect of the body schema on body sway by balance- or coordination-related sport types, body sway might be further influenced by other types of sports as well, such as strength training and endurance training, since muscle strength especially of the legs influences postural control and the risk of falling^20^. Future studies should include the type of sports already systematically for recruiting participants. Especially recruiting individuals who participate in coordination- and balance-relevant sports (e.g. expert dancers or martial artists) or strength and endurance sports (e.g. wrestlers or cyclists) might give further insights to when body representations, especially body schema, affect postural control. Moreover, the role of the covert body schema task performance on body sway could be further investigated. However, since the overt body schema measure resulted to be more relevant for explaining postural control, future studies should rather focus on further investigating the influence of the *Taking a Posture* task on postural control in different population groups, such as mentioned before, but also e.g. in different patient groups, such as stroke patients, but also individuals with body image distortions, such as anorexic, bulimic, obese, or even individuals with a continuous or sudden change of body shape or weight, such as e.g. in pregnant women or after surgery. Finally, future studies including more subjects to increase the power for the individual independent variables, and systematically manipulating indirect high-order body representation variables while maintaining other factors constant are required for a solid cause-effect conclusion.

## Conclusion

This is the first study that directly links sex/gender differences in the control of body balance to the role of high-order body representations in postural control. We propose two types of measures to assess the body schema: once from motor control side including feedback of the body’s configuration in space generated by movements (overt task), and another from motor cognition side including an internal simulation without receiving movement-generated feedback (covert task: Laterality task (*LAT*)). To assess the body schema by an overt whole body task, we propose a *Taking-a-Posture* task (*TaP*) in which participants are asked to repeatedly take certain postures of different complexities. Body schema as well as subjective evaluation of the own body related to concurrent sensorimotor control of body sway. However, body image dissatisfaction was found to be only associated with postural control in females, but not in males. Finally, we have discussed how high-level representations of the self, both body image and body schema, may be involved in predictive motor/postural control. This hypothesis-generating proof-of-concept study lays the basis for future studies, which should include conditional process analysis and causal inference for a better understanding of the underlying mechanisms.

## Methods

### Subjects

42 healthy young adults (21 females; 18–35 years) participated in this study (detailed inclusion criteria in Supplementary). Given an anticipated medium to strong effect size (Cohen’s f$^2$ = 0.32) for the influence of height on root-mean-square CoP medio-lateral (ML)^7^, and an expected power of 0.9 with a total number of 9 predictors a total sample of at least 36 subjects was required (a-priori, g-power). All participants gave written informed consent. The investigation was carried out in accordance with the principles of the Declaration of Helsinki and was approved by the medical ethical committee of the Technical University of Munich (248/19 S-SR). Demographics and characteristics of included subjects are shown in Tables 1 and 2 as well as in the Supplementary Tables S1 and S2.

### Experimental procedure

**Figure 4:**
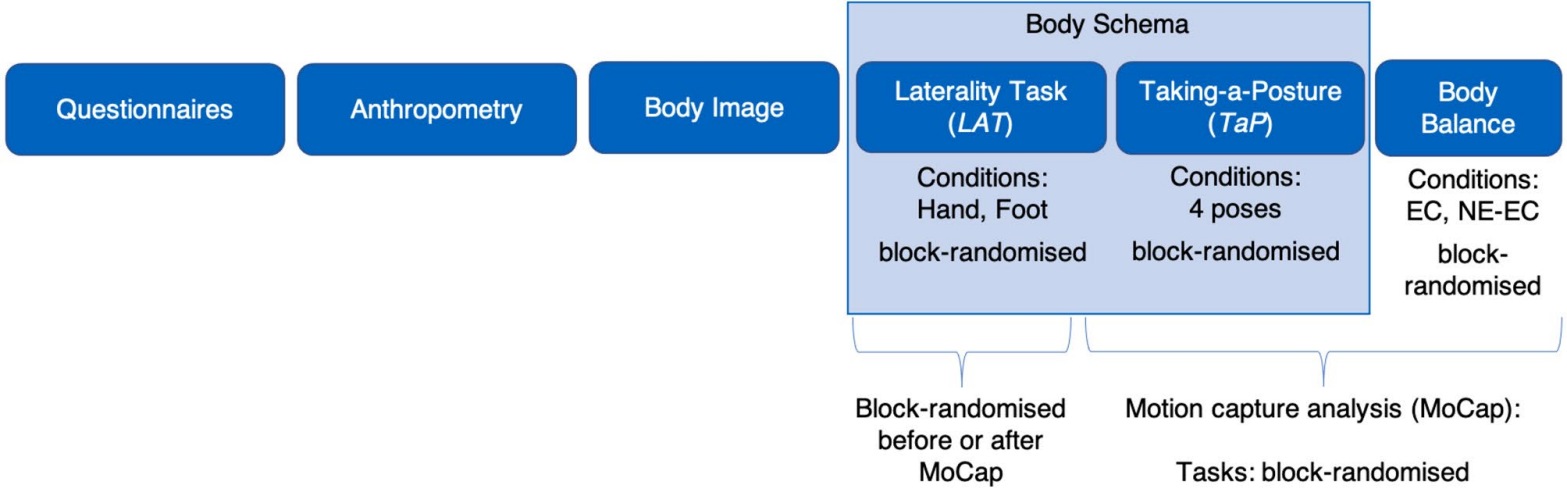
Experimental procedure. Balance: bipedal quiet standing with eyes closed (EC) and neck extended (NE-EC).

The experimental procedure is shown in Fig. 4. We first assessed possible exclusion criteria and factors possibly influencing balance or body representations via Psytoolkit^73,74^ (such as sports experience, mirror usage, dealing with body form, profession^75^, as well as self-esteem (RSES)^76^, physical appearance (PACS)^77^, and drive for muscularity (DMS)^78^). Furthermore, we assessed the handedness and footedness (preferred leg for single-legged stance, and shooting leg). For the lab visit, participants were asked to wear tight, non-reflective, dark clothes.

During lab visit, participants were asked to approve their previously given answers and adapt them if needed. Informed consent was obtained to publish images in publications. Anthropometric measures were taken without shoes. *Body image* (BI) was assessed by the Body Image Assessment Score - Body Distortion (BIAS-BD) figural drawing^79^. To assess *body schema* (BS), we used an overt and a covert task.

**Figure 5:**
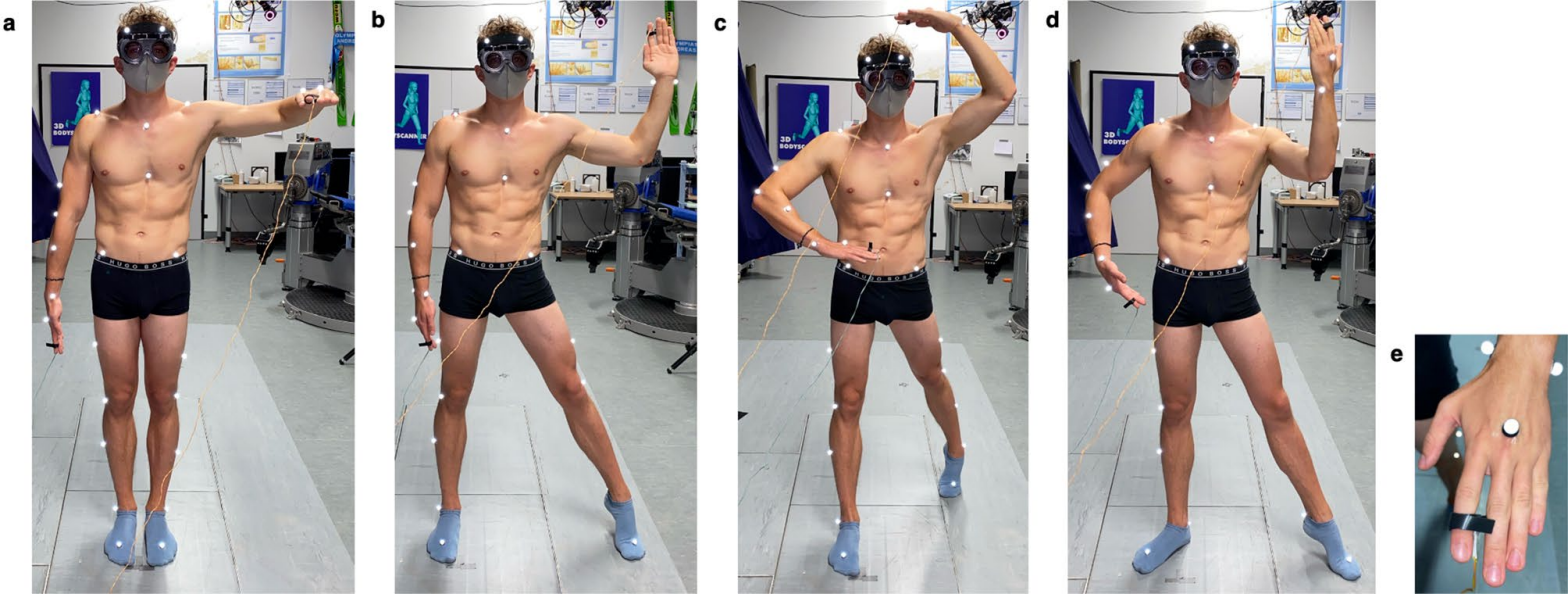
*Taking-a-pasture* task: four poses of different complexity (change in numbers of limbs involved) with Vicon full-body plugin-gait marker set and goggles to restrict vision of own body. (**a**) One upper extremity (non-dominant); (**b**) one upper and one lower extremity (both non-dominant); (**c**) two upper extremities and one lower extremity (non-dominant: arm above head); (**d**) all extremities explicitly involved in posture (non-dominant: arm facing head, foot extended); (**e**) button placement.

The overt *Taking-a-posture* (*TaP*) task, consisted of four target poses with different numbers of extremities actively involved to allow for different level of complexity (Fig. 5a–d) , from one upper limb (non-dominant), via one upper and one lower limb (both non-dominant), and two upper limbs and one lower limb (non-dominant: arm above head) to the most complex posture with all limbs explicitly involved in the posture (non-dominant: arm facing head, foot extended).The hand and foot positions and orientations were inspired by previous works in the field of apraxia and imitation (e.g.^80,81^). Participants were first shown one of these poses and asked to take the approximate posture, which was then refined by the investigator using a goniometer. Subjects were asked to remember the refined position (reference posture), and press any of the two buttons attached to the index fingers (Fig. 5), as soon as the investigator indicated so ($\sim$4s post-correction). In-between trials subjects took a neutral posture (upright quiet standing) for 15s. The task then was to retake the reference posture as quickly and as accurately as possible and press any of the two buttons when having reached the felt correct position, and maintain that for $\sim$4s. After a first familiarisation trial, five test trials were conducted. This procedure was repeated for all four postures. Since the non-dominant side performs better in proprioceptive tasks^82^, the focus of the *TaP* task was the non-dominant hand and foot (opposite to shooting leg) (Fig. 5).

**Figure 6:**
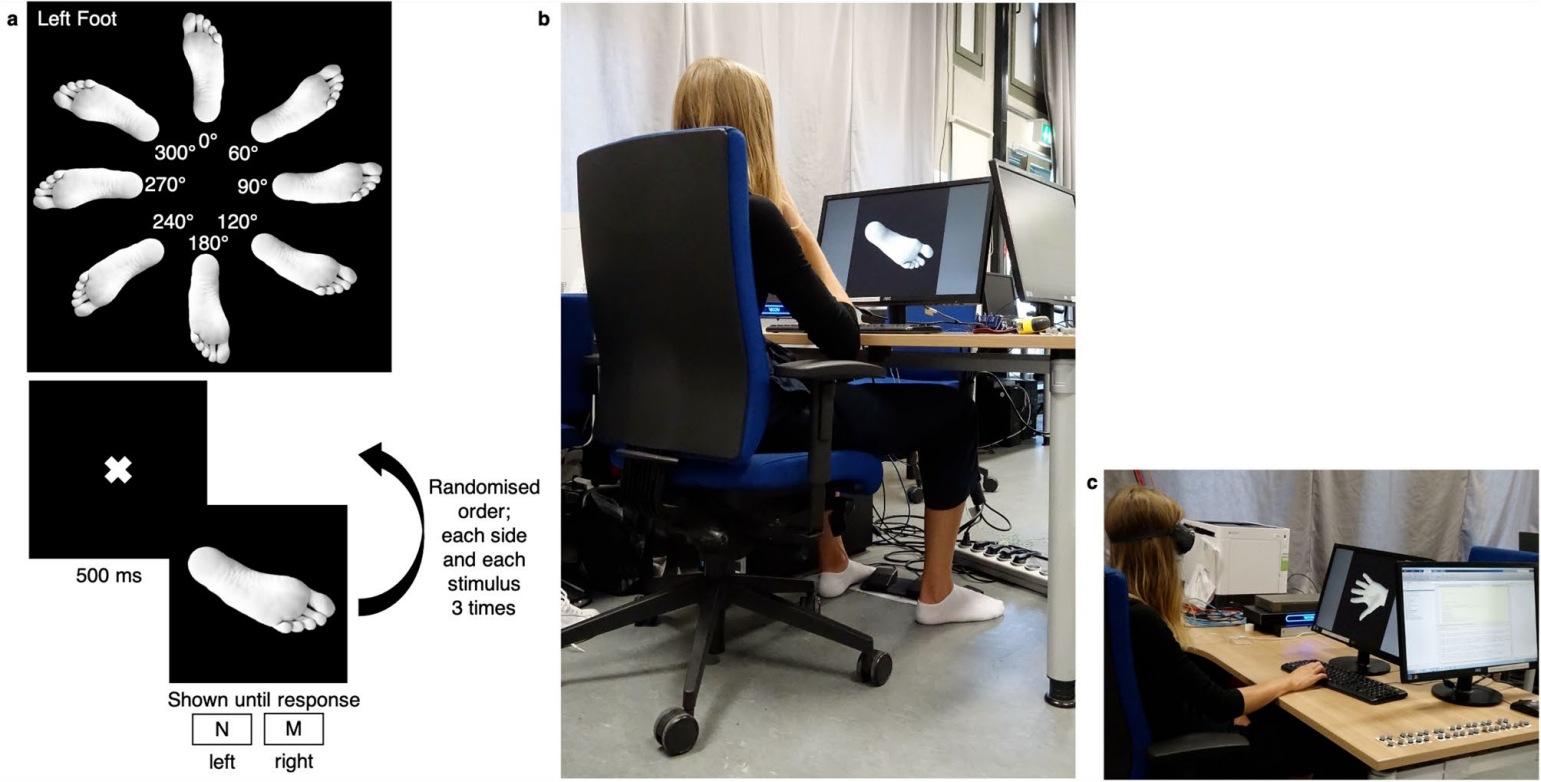
*Laterality* task; (**a**) top: 8 orientations/stimuli presented, examplary for *FLAT*; a bottom: task procedure, N and M represent the keyboard keys to press for *HLAT*, which corresponded to left and right pedal for *FLAT*; (**b**) setup during *FLAT*; (**c**) setup during *HLAT*.

The covert task consisted of the *Laterality task* (*LAT*) for hands (*HLAT*) and feet (*FLAT*)^75^. The stimuli for the *LAT* task contained gender-matched gray-scale pictures^83^/mirror-pictures^68^ of the left or right hand/foot. To stimulate motor imagery rather than visuo-spatial rotation of the image^84^, hand palms and foot soles were presented. 48 trials^69^ (2 sides x 8 rotations x 3 trials (Fig. 6a) were conducted for each condition (*HLAT*/*FLAT*), after a short familiarisation phase (6 randomised trials), respectively. Rotations consisted of 0$^\circ$, 60$^\circ$, 120$^\circ$, 180$^\circ$, 240$^\circ$, 300$^\circ$^69,84^, and 90$^\circ$ and 270$^\circ$ for comparability with medio-lateral intrinsic joint constraints^68^ (i.e. future use of data). Stimuli were presented with Matlab using Psychtoolbox (Psychophysics Toolbox Version 3 (PTB-3)). In the *FLAT* condition (Fig. 6b), participants were asked to press the left or right pedal with their respective foot. For the *HLAT* condition (Fig. 6c), subjects were asked to press “N” and “M” on the keyboard, respectively, with their index and middle finger of their preferred hand^84^. To restrict visual input of their own body, participants wore goggles for the *HLAT* and the *TaP* task.

Body sway was assessed during quiet standing (35s) in a narrow upright bipedal stance with a 2.5cm inter-foot distance with eyes closed (EC) and arms hanging relaxed on the sides. A narrow foot position (e.g.^4,35,85^) was chosen for inducing a more challenging stance than hip-wide stance^86,87^ and simultaneously reduce the contact area between extremities for less cutaneous feedback. We conducted two conditions: with/without neck extended (NE), respectively: NE-EC, EC (Supplementary Fig. S1). Extending the neck was chosen to make the stance condition more difficult without changing the BoS due to reduced sensitivity of the vestibular channel^10^, which might increase the reliance on proprioceptive and tactile information. Moreover, it has been reported that vestibular disturbances do not alter the internal body representation of verticality^88,89^. Participants were asked to perform each balance condition six times (1 familiarisation trial, 5 test trials) with a 30s break between trials. For hygiene reasons subjects wore own thin summer socks. Due to the thin material and smooth texture, no substantial affects due to sock texture were expected^90^.

### Measurement devices

For motion analysis, an optoelectronic motion capture (MoCap) system (Vicon Nexus 2.9; 200Hz) with 10 cameras (9 Bonitas/Veros, 1 DV camera) and a force plate are used (Advanced Mechanical Technology, Inc. (AMTI), Watertown, MA; 1000Hz). The buttons used in the *TaP* task are used for event-triggering within Vicon when the final posture is reached. The MoCap system is calibrated at the beginning of the *TaP* task by a static and dynamic calibration, and by another static calibration before the balance task. The force plate is calibrated (auto-zeroed) in an unloaded state between conditions in the *TaP* task and before each balance trial.

### Data processing and parameter definition

Body image distortion (BID) and body image dissatisfaction (BIDS) were calculated, based on the differences between the real (R), estimated (A), and ideal BMI (I)^79^. The absolute values of the mean ($\mu$) of the two trials (n) (Eqs. 1, 2) were used for statistical analysis.

1 $$\begin{aligned}&y_{BID} = \left|\mu _{i = 1}^{n}(\tfrac{ (x_{A_i}-x_{R_i})}{x_{R_i}}\cdot 100)\right| \end{aligned}$$

2 $$\begin{aligned}&y_{BIDS} = \left|\mu _{i = 1}^{n}(\tfrac{ (x_{I_i}-x_{A_i})}{x_{A_i}}\cdot 100)\right| \end{aligned}$$

For the *TaP* task, data were first post-processed within Vicon software (10 Hz Woltring Filter). Then, the angle error per joint was calculated, averaged across the total number of trials ($N_{i}$), based on which the mean ($\mu$) (Eq. 3) and standard deviation (SD $\sigma$) (Eq. 4) across the total number of poses ($N_{p}$) were computed. $x_{Imi_i}$ are the joint angles of the imitation trials (i). $x_{Ref_p}$ are the joint angles of the reference trials of each pose (*p*). $N_{c}$ is the total number of Cartesian coordinates (*c*), here three: in x, y, and z. $N_{j}$ is the total number of joints *j*, here 24. To obtain positive values for a better performance, results were inverted at the end.

3 $$\begin{aligned}&y_{{TaP} \,accuracy} = \mu _{p = 1}^{N_p}\left( \mu _{i = 1}^{N_i}\left( \tfrac{\sum _{j = 1}^{N_j}(\sum _{c = 1}^{N_c}|(x_{Imi_i}-x_{Ref_p})|)}{N_i\cdot N_c}\right) \right) \cdot (-1) \end{aligned}$$

4 $$\begin{aligned}&y_{{TaP}\, variability} = \mu _{p = 1}^{N_p}\left( \sigma _{i = 1}^{N_i}\left( \tfrac{\sum _{j = 1}^{N_j}(\sum _{c = 1}^{N_c}|(x_{Imi_i}-x_{Ref_p})|)}{N_i\cdot N_c}\right) \right) \cdot (-1) \end{aligned}$$

For the *LAT* task, we calculated the average across extremities as an overall laterality parameter for response accuracy (*LAT* accuracy) and response latency (*LAT* latency), respectively.

Centre of pressure (CoP) data were post-processed by a Matlab-routine in the following steps: (1) 10 Hz low-pass Butterworth-filtered, (2) differentiated one-dimensional (1D) and two-dimensional (2D) parameters and (3) extracted 1D and 2D parameters. Within this work, we focused on the body sway parameter SD CoP as a time-independent sway parameter, previously used in other studies^11,12,14^ which has also been reported as well reliable parameter for discriminating different groups^88^. Furthermore, for time-dependent parameter we used the stabilogram diffusion analysis (SDA)^35^, which describes the CoP trajectories as fractional Brownian motion. Collins and De Luca^35^ have proposed the SDA to provide information about both the feedforward and feedback control as the two control strategies. On the one hand, the short-term intervals ($\Delta$t < 1s) are discussed to be dominated by open-loop control due to predominantly positively correlated CoP trajectories and thus a persistent behaviour (Hurst exponent $\hbox {H}_{\mathrm{s}} > 0.5$). On the other hand, the long-term intervals (1 < $\Delta$t < 10 s) represent the feedback control due to predominantly negatively correlated CoP trajectories and thus an anti-persistent behaviour ($\hbox {H}_{\mathrm{l}} < 0.5$). However, the SDA has been criticised to underestimate long-term correlations^91^ due to fractional Brownian motions being unbounded, and other approaches to estimate the long-term Hurst exponent have been proposed, such as e.g. the Detrended Fluctuation Analysis. In this study we focus on the analysis of the short-term region extracted by the SDA to connect body representations measures with feed-forward processes during body sway. While the scaling exponent H describes the correlation of consecutive CoP displacements, the diffusion coefficient ($\hbox {D}_{\mathrm{s}}$) represents the degree of stochastic activity, a higher value indicating a higher stochastic activity and thus a drift away from the equilibrium. A higher stochastic activity also indicates more exploratory behaviour^16^. Finally, the transition point (TP; coordinates [${TP}_{{t}}$, ${TP}_{{d}}$]) represents the switching point between feed-forward (open-loop/short-term intervals) and feedback (closed-loop/long-term intervals) control, whereby an exceeding of the feedback threshold at lower time intervals indicates an earlier switch to feedback-control in terms of time and a lower threshold indicates a tighter controlled sway area. Since the diffusion coefficients have previously reported to have a higher inter-individual variability than the Hurst exponent, and was able to discriminate well different groups and sensory conditions^92–94^, ${D}_{{s}}$ was used for further statistical analysis, while all SDA parameters are reported in the descriptive results.

### Statistical analysis

Due to missing data in two subjects, one univariate outlier (>3SD) in body weight, and three subjects being multivariate outliers (Cook’s Distance (*D)*> 3*$\mu$; $\mu$ = mean *D*), descriptive and statistical analysis were computed for 36 subjects (19 females).

Hierarchical multiple linear regressions were computed for body sway (SD CoP) with six steps: (1) sensory condition (SC: EC; NE-EC), (2) gender (male; female), (3) age, (4) anthropometry (Anthr.: height; weight), (5) body schema (BS: *TaP*_v_; *LAT*_a_), (6) body image (BI: BIDS_abs_; BID_abs_). In the whole group further gender-interactions were added in separate steps for height (H_gen_), weight (W_gen_), *TaP*_v_ (TaP_gen_), *LAT*_a_ (LAT_gen_), BIDS_abs_ (BIDS_gen_) and BID_abs_ (BID_gen_). For this, gender was dummy coded once coding females as 1 and males as 0 (f), and once coding males as 1 and females as 0 (m). The interaction variables were then computed once for males and once for females by the product of the dummy variable and the other interaction variable; e.g. in the regression step of BIDS both I-BIDS(m) and I-BIDS(f) were added. The order of the steps has been chosen from low-level to high-level control and from intra-individual to inter-individual factors. Gender and age are demographic influencing factors. Since we expected age to influence inter-individual variability less in our sample, gender was added first. Demographics was added before anthropometry, since latter can vary within a certain gender or age group.

In a next step we computed backward multiple linear regressions (exclusion if *P* > 0.1) using the overall models to find the best regression models with minimal number of explanatory variables. Normal distribution was given for all regression models (Shapiro-Wilk of residuals: *P* > 0.05; and visual inspection of normal P-P-plot). Explanatory variables (explanans/explanantia) were independent of each other so that multicollinearity was not observed (r<0.7; variable inflation factor (VIF)<10; Tolerance > 0.1). Homoscedascity was also given in all regression models for SD CoP (visual inspection of scatter plots of standardised predicted values and standardised residuals; Breusch Pagan test: *P* > 0.05). If homoscedascity was not given, the regression model was corrected by using robust standard errors and corrected t- and p-values are reported (t_a_ and $${P}_{{a}}$$, respectively). Standardised regression coefficients were then calculated by multiplying the unstandardised regression coefficient B corrected by the robust standard error with the division of the standard deviation of the independent variable by the standard deviation of the dependent variable. Due to exploratory nature and comparability reasons across gender and the whole group, variables were also included, even though they did not show a significant linear relationship with body sway. The amount of the variance explained by an individual variable when controlling for the others is reported by the squared partial correlation (*pr* $$^2$$). Effect sizes are reported as Cohen’s *f*$$^2$$ (small: *f*$$^2$$ = 0.02, medium: *f*$$^2$$ = 0.15, large: *f*$$^2$$ = 0.35)^95^. Finally, post-hoc statistical power was calculated with G-power^96^ for each independent variable and is reported as *SP*. *pr* $$^2$$, *f*$$^2$$ and *SP* are only reported for the best (backward) models. In case that homoscedascity was violated, effect sizes are reported as partial squared eta *p*$$\eta $$$$^2$$ (small: *p*$$\eta $$$$^2$$ = 0.01, medium: *p*$$\eta $$$$^2$$ = 0.06, large: *p*$$\eta $$$$^2$$ = 0.14) and observed statistical power *oSP*. A statistical power of 0.8 was considered as good.

### Ethics declaration

This study was carried out in accordance with the principles of the Declaration of Helsinki and was approved by the medical ethical committee of the Technical University of Munich (248/19 S-SR). All participants gave written informed consent.

## Supplementary Information

Supplementary Information.

## Data Availability

The datasets generated during and/or analysed during the current study are available from the corresponding authors on reasonable request.
