# Supplementary material for: Sex differences in the association of postural control with indirect measures of body representations
Source: Sci Rep. 2022 Mar 16;12:4556. doi: 10.1038/s41598-022-07738-8 (PMC8927351; doi:10.1038/s41598-022-07738-8)
Supplement: Supplementary file 1 — Supplementary Information. [file 41598_2022_7738_MOESM1_ESM.pdf]

Supplementary material for Sex differences in the association of postural control with indirect measures of body representations

Katrin H. Schulleri[1 *], Leif Johannsen[2,3 *], Youssef Michel[1], and Dongheui Lee[1,4]

[1]Technical University of Munich (TUM), Department of Electrical and Computer Engineering (EI), Human-centered Assistive Robotics (HCR), Munich, 80333, Germany
[2]RWTH Aachen University, Institute of Psychology, Cognitive and Experimental Psychology, Aachen, 52066, Germany
[3]Technical University of Munich (TUM), Department of Sport and Health Sciences, Human Movement Science, 80992, Munich, Germany
[4]German Aerospace Center (DLR), Institute of Robotics and Mechatronics, Weßling, 82234, Germany
[*]katrin.schulleri@tum.de, leif.johannsen@psych.rwth-aachen.de

Competing interests statement

The authors declare that the research was conducted in the absence of any commercial or financial relationships that could be construed as a potential conflict of interest.

Inclusion/exclusion criteria

Participants were included if they reported no: current psychological/psychiatric disorders, acute eating disorders, disorders of the central nervous or vestibular system with increasing risk of falling or stance instability, dizziness, polyneuropathy, drug or alcohol abuse, current medication that might influence body sway (e.g. opiate, antidepressants, hypnotics, sedatives) and pain while standing.

Methods - additional correlation analysis

In addition to the main analysis, we computed correlation analysis for hypothesis generation. If data were normally distributed (Shapiro-Wilk test>0.05) Pearson's correlation coefficients were observed (Supplementary Tables S3-S5, otherwise Kendall's tau correlation coefficients.

Supplementary tables and figures

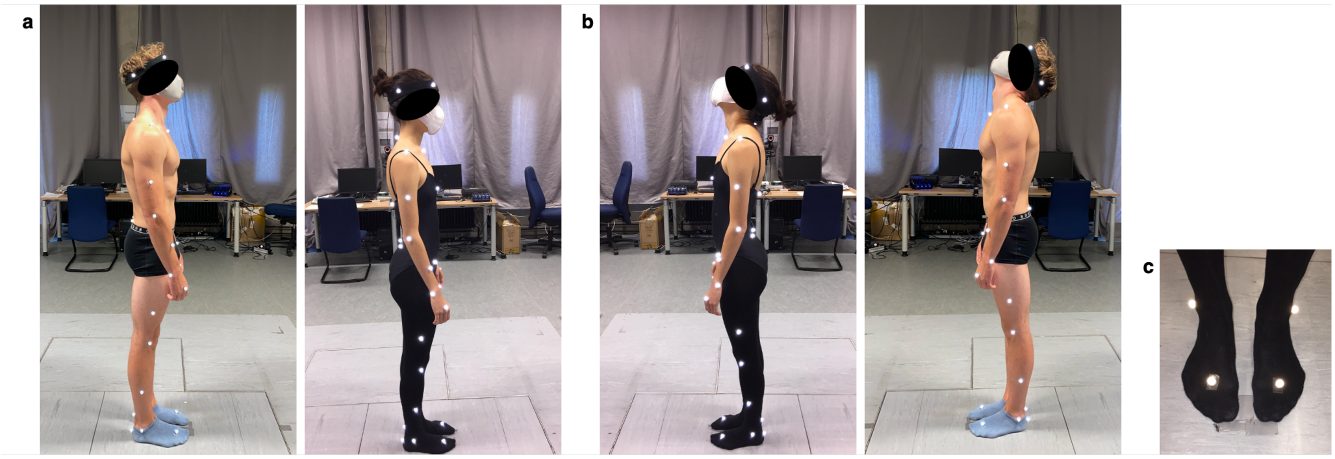

Figure S1. Setup of the balance task: bipedal quiet standing with eyes closed (EC) (a) and neck extended (NE-EC) (b); marked foot position (2.5cm inter-foot distance).

Table S1. Subject characteristics of handedness, footedness (stance leg), BMI category, and sports-related variables; number of participants (percentage of all participants of the respective group).

Variables	Whole group (n=36)	Males (n=17)	Females (n=19)
**Handedness**			
left	2 (5.6%)	1 (5.9%)	1 (5.3%)
right	34 (94.4%)	16 (94.1%)	18 (94.7%)
**Footedness**			
left	9 (24.32%)	6 (35.3%)	2 (10.5%)
right	21 (56.76%)	8 (47.1%)	13 (68.4%)
both	7 (18.92%)	3 (17.6%)	4 (21.05%)
**BMI Category**			
underweight: <18.5	2 (5.6%)	0 (0.00%)	2 (10.5%)
normal: 18.5-24.9	30 (83.3%)	14 (82.4%)	16 (84.2%)
overweight: >25	4 (11.1%)	3 (17.6%)	1 (5.3%)
**Sports-Related Variables**			
Regular Sports	34 (94.4%)	16 (94.1%)	18 (94.7%)
Cardio	25 (69.4%)	13 (76.5%)	12 (63.2%)
Team	10 (27.8%)	5 (29.4%)	5 (26.3%)
Resistance	20 (55.6%)	8 (47.1%)	12 (63.2%)
Coordination	12 (30.6%)	3 (17.6%)	8 (42.1%)
Importance of Shape in Sports	14 (38.9%)	8 (47.1%)	6 (31.6%)

.

Table S2. Subject characteristics of variability of sway velocity and long-term stabilogram diffusion parameter and group comparisons (t-tests and Mann-Whitney-U tests)

Variables	Whole group (n=36) mean ± SD	Males (n=17) mean ± SD	Females (n=19) mean ± SD	Significance (gender comparison)
**Body Sway**				
SD dCoP (mm/s)	21.39 ± 5.33	21.57 ± 4.19	21.23 ± 6.23	0.57
in EC	19.89 ± 4.07	19.77 ± 2.49	20.00 ± 5.17	0.87
in NE-EC	22.88 ± 6.03	23.37 ± 4.80	22.45 ± 7.06	0.53
D_l (mm^2/s)	3.97 ± 2.66	3.98 ± 2.68	3.95 ± 2.68	0.46
in EC	3.60 ± 2.52	3.49 ± 2.88	3.70 ± 2.24	0.81
in NE-EC	4.34 ± 2.78	4.47 ± 2.46	4.21 ± 3.10	0.79
H_l (mm^2/s)	0.12 ± 0.08	0.12 ± 0.09	0.12 ± 0.07	0.71
in EC	0.13 ± 0.08	0.12 ± 0.09	0.14 ± 0.07	0.61
in NE-EC	0.11 ± 0.08	0.11 ± 0.09	0.11 ± 0.08	0.97

.

Table S3. Correlation coefficients (Pearson and Kendall's Tau) of variables in whole group.

Var.	Age	H	W	PACS	DMS	RSES	$BIDS_{abs}$	BID_{abs}	$BIDS_{rel}$	BID_{rel}	Fr.Sp.	TaP_v	LAT_a	SD CoP	D_s	H_s	TP_t	H_l	D_l
Age	1	0.12	**0.21**	-0.02	-0.02	0.17	**-0.20**	**0.22**	0.06	0.14	**0.35**	**0.25**	-0.11	-0.15	-0.13	0.08	-0.13	0.02	-0.14
H	0.12	1	**0.56**	0.01	0.09	**-0.22**	-0.09	0.13	0.08	0.11	-0.02	-0.09	0.09	**0.26**	**0.16**	0.18	0.16	0.01	0.22
W	**0.21**	**0.56**	1	0.15	**0.23**	0.04	-0.01	0.03	-0.04	-0.10	0.02	-0.08	**0.21**	**0.30**	**0.16**	0.15	0.09	**-0.26**	0.05
PACS	-0.02	0.01	0.15	1	0.12	-0.02	**0.23**	0.14	-0.14	0.06	-0.03	**0.19**	-0.12	-0.12	-0.07	**-0.21**	-0.02	-0.06	**-0.21**
DMS	-0.02	0.09	**0.23**	0.12	1	0.04	0.04	**-0.24**	0.11	**- 0.31**	0.05	**-0.17**	0.10	**0.19**	0.11	**0.21**	0.00	0.03	0.12
RSES	0.17	**-0.22**	0.04	-0.02	0.04	1	0.12	-0.09	-0.12	-0.05	0.05	0.02	-0.03	-0.11	-0.12	0.06	**-0.22**	0.00	-0.02
$BIDS_{abs}$	**-0.20**	-0.09	-0.01	**0.23**	0.04	0.12	1	-0.01	**-0.46**	0.03	-0.04	-0.02	0.14	0.10	0.13	0.02	-0.06	-0.14	-0.01
BID_{abs}	**0.22**	0.13	0.03	0.14	**-0.24**	-0.09	-0.01	1	**-0.22**	**0.76**	0.12	**0.24**	-0.13	-0.12	-0.02	-0.02	-0.04	0.03	0.00
$BIDS_{rel}$	0.06	0.08	-0.04	-0.14	0.11	-0.12	**-0.46**	**-0.22**	1	**-0.34**	0.15	**0.16**	**-0.17**	-0.15	**-0.25**	0.02	0.02	0.12	-0.08
BID_{rel}	0.14	0.11	-0.10	0.06	**-0.31**	-0.05	0.03	**0.76**	**-0.34**	1	0.02	**0.16**	-0.06	-0.07	-0.00	-0.04	-0.07	0.04	0.08
Fr.Sp.	**0.35**	-0.02	0.02	-0.03	0.05	0.05	-0.04	0.12	0.15	0.02	1	**0.32**	-0.13	**-0.42**	**-0.40**	0.04	**-0.21**	0.15	-0.16
TaP_v	**0.25**	-0.09	-0.08	**0.19**	**-0.17**	0.02	-0.02	**0.24**	**0.16**	**0.16**	**0.32**	1	**-0.30**	**-0.40**	**-0.31**	0.04	**-0.20**	0.10	**-0.19**
LAT_a	-0.11	0.09	**0.21**	-0.12	0.10	-0.03	0.14	-0.13	**-0.17**	-0.06	-0.13	**-0.30**	1	**0.26**	**0.24**	-0.03	**0.16**	**-0.19**	0.02
SD CoP	-0.15	**0.26**	**0.30**	-0.12	**0.19**	-0.11	0.10	-0.12	-0.15	-0.07	**-0.42**	**-0.40**	**0.26**	1	**0.64**	0.10	**0.30**	**-0.36**	**0.32**
D_s	-0.13	**0.16**	**0.16**	-0.07	0.11	-0.12	0.13	-0.02	**-0.25**	-0.00	**-0.40**	**-0.31**	**0.24**	**0.64**	1	**0.16**	**0.16**	**-0.36**	**0.17**
H_s	0.08	0.18	0.15	**-0.21**	**0.21**	0.06	0.02	-0.02	0.02	-0.04	0.04	0.04	-0.03	0.10	**0.16**	1	**-0.58**	0.11	**0.24**
TP_t	-0.13	0.16	0.09	-0.02	0.00	**-0.22**	-0.06	-0.04	0.02	-0.07	**-0.21**	**-0.20**	**0.16**	**0.30**	**0.16**	**-0.58**	1	**-0.34**	0.01
H_l	0.02	0.01	**-0.26**	-0.06	0.03	0.00	-0.14	0.03	0.12	0.04	0.15	0.10	**-0.19**	**-0.36**	**-0.36**	0.11	**-0.34**	1	**0.56**
D_l	-0.14	0.22	0.05	**-0.21**	0.12	-0.02	-0.01	0.00	-0.08	0.08	-0.16	**-0.19**	0.02	**0.32**	**0.17**	**0.24**	0.01	**0.56**	1

H=height; W=weight; Fr.Sp.=FreqSports; TaP_v=TaP variability; LAT_a=LAT accuracy; D_s=short-term diffusion coefficient, D_l=long-term diffusion coefficient, H_s=short-term Hurst exponent, $_l$=long-term Hurst exponent, TP_t=transition time point
Bold indicates significance ($p \leq 0.05$).

Table S4. Correlation coefficients (Pearson and Kendall's Tau) of variables in males.

Var.	Age	H	W	PACS	DMS	RSES	$BIDS_{abs}$	BID_{abs}	$BIDS_{rel}$	BID_{rel}	Fr.Sp.	TaP_v	LAT_a	SD CoP	D_s	H_s	TP_t	H_l	D_l
Age	1	0.13	**0.34**	-0.04	-0.15	-0.22	-0.09	0.19	-0.09	0.24	0.22	0.06	-0.02	0.03	0.08	0.12	-0.14	-0.30	**-0.40**
H	0.13	1	0.02	**-0.33**	-0.31	**-0.29**	-0.16	0.10	0.03	**0.34**	0.05	-0.01	-0.07	0.16	-0.13	0.14	0.01	**0.43**	**0.43**
W	**0.34**	0.02	1	0.18	0.00	0.02	-0.13	0.21	**-0.26**	0.07	**0.29**	0.15	-0.13	0.06	0.14	0.19	-0.10	-0.06	0.03
PACS	-0.04	**-0.33**	0.18	1	**0.28**	0.20	**0.47**	**0.35**	**-0.37**	0.13	0.09	0.18	-0.22	-0.14	-0.04	0.02	-0.20	-0.03	-0.02
DMS	-0.15	-0.31	0.00	**0.28**	1	0.08	0.05	-0.10	-0.13	-0.23	-0.12	-0.07	-0.16	0.13	0.08	0.33	0.02	0.27	**0.37**
RSES	-0.22	**-0.29**	0.02	0.20	0.08	1	0.14	0.01	-0.03	-0.08	-0.04	0.16	-0.20	-0.17	-0.04	0.12	**-0.36**	-0.02	-0.08
$BIDS_{abs}$	-0.09	-0.16	-0.13	**0.47**	0.05	0.14	1	0.05	-0.13	0.02	0.08	0.13	0.09	-0.12	-0.04	-0.03	-0.08	-0.08	-0.04
BID_{abs}	0.19	0.10	0.21	**0.35**	-0.10	0.01	0.05	1	-0.20	**0.57**	**0.30**	**0.44**	**-0.24**	-0.12	-0.04	-0.04	-0.10	-0.02	-0.05
$BIDS_{rel}$	-0.09	0.03	**-0.26**	**-0.37**	-0.13	-0.03	-0.13	-0.20	1	**-0.44**	0.03	0.17	-0.19	-0.16	**-0.30**	0.08	-0.01	0.04	-0.14
BID_{rel}	0.24	**0.34**	0.07	0.13	-0.23	-0.08	0.02	**0.57**	**-0.44**	1	0.16	0.19	0.01	0.17	0.00	-0.12	-0.03	-0.10	0.02
Fr.Sp.	0.22	0.05	**0.29**	0.09	-0.12	-0.04	0.08	**0.30**	0.03	0.16	1	**0.34**	-0.05	**-0.42**	**-0.41**	-0.15	-0.15	0.13	-0.14
TaP_v	0.06	-0.01	0.15	0.18	-0.07	0.16	0.13	**0.44**	0.17	0.19	**0.34**	1	**-0.36**	**-0.29**	**-0.24**	0.09	-0.20	0.14	0.02
LAT_a	-0.02	-0.07	-0.13	-0.22	-0.16	-0.20	0.09	**-0.24**	-0.19	0.01	-0.05	**-0.36**	1	0.08	0.19	-0.23	**0.24**	-0.18	-0.09
SD CoP	0.03	0.16	0.06	-0.14	0.13	-0.17	-0.12	-0.12	-0.16	0.17	**-0.42**	**-0.29**	0.08	1	**0.61**	0.15	**0.34**	-0.28	**0.35**
D_s	0.08	-0.13	0.14	-0.04	0.08	-0.04	-0.04	-0.04	**-0.30**	0.00	**-0.41**	**-0.24**	0.19	**0.61**	1	0.16	0.19	**-0.43**	0.14
H_s	0.12	0.14	0.19	0.02	0.33	0.12	-0.03	-0.04	0.08	-0.12	-0.15	0.09	-0.23	0.15	0.16	1	**-0.55**	0.27	**0.34**
TP_t	-0.14	0.01	-0.10	-0.20	0.02	**-0.36**	-0.08	-0.10	-0.01	-0.03	-0.15	-0.20	**0.24**	**0.34**	0.19	**-0.55**	1	**-0.39**	-0.04
H_l	-0.30	**0.43**	-0.06	-0.03	0.27	-0.02	-0.08	-0.02	0.04	-0.10	0.13	0.14	-0.18	-0.28	**-0.43**	0.27	**-0.39**	1	**0.68**
D_l	**-0.40**	**0.43**	0.03	-0.02	**0.37**	-0.08	-0.04	-0.05	-0.14	0.02	-0.14	0.02	-0.09	**0.35**	0.14	**0.34**	-0.04	**0.68**	1

H=height; W=weight; Fr.Sp.=frequency of sports; TaP_v=*TaP* variability; LAT_a=LAT accuracy; D_s=short-term diffusion coefficient, D_l=long-term diffusion coefficient, H_s=short-term Hurst exponent, $_l$=long-term Hurst exponent, TP_t=transition time point

Bold indicates significance ($p \leq 0.05$)

Table S5. Correlation coefficients (Pearson and Kendall's Tau) of variables in females.

Var.	Age	H	W	PACS	DMS	RSES	$BIDS_{abs}$	BID_{abs}	$BIDS_{rel}$	BID_{rel}	Fr.Sp.	TaP_v	LAT_a	SD CoP	D_s	H_s	TP_t	H_l	D_l
Age	1	-0.09	0.09	-0.02	-0.08	-0.19	**-0.42**	0.31	**0.24**	0.20	**0.45**	**0.51**	**-0.24**	**-0.40**	**-0.32**	0.11	-0.16	0.23	-0.09
H	-0.09	1	**0.47**	0.18	0.04	**-0.26**	-0.26	**0.25**	0.02	0.27	-0.16	-0.16	0.10	0.21	**0.37**	0.20	0.29	-0.19	0.12
W	0.09	**0.47**	1	0.20	0.05	-0.01	-0.11	0.21	-0.07	0.12	-0.16	-0.01	**0.36**	0.10	0.17	0.04	**0.23**	-0.17	0.03
PACS	-0.02	0.18	0.20	1	0.01	**-0.25**	-0.03	0.03	0.12	0.02	-0.15	**0.25**	0.00	-0.14	-0.02	**-0.28**	0.12	-0.12	**-0.35**
DMS	-0.08	0.04	0.05	0.01	1	0.08	-0.11	**-0.25**	0.22	**-0.26**	0.09	-0.13	**0.23**	**0.23**	0.11	0.21	0.01	-0.04	0.03
RSES	-0.19	**-0.26**	-0.01	**-0.25**	0.08	1	0.13	-0.18	-0.19	-0.07	0.14	-0.07	0.07	-0.08	-0.23	0.03	-0.11	0.00	-0.05
$BIDS_{abs}$	**-0.42**	-0.26	-0.11	-0.03	-0.11	0.13	1	-0.08	**-0.78**	0.12	-0.19	-0.12	-0.17	**0.43**	**0.30**	0.10	-0.22	**-0.37**	-0.03
BID_{abs}	0.31	**0.25**	0.21	0.03	**-0.25**	-0.18	-0.08	1	-0.15	**0.90**	0.01	0.14	-0.04	-0.10	-0.04	0.01	-0.00	0.11	0.10
$BIDS_{rel}$	0.24	0.02	-0.07	0.12	0.22	-0.19	**-0.78**	-0.15	1	-0.19	**0.30**	0.14	-0.13	**-0.25**	**-0.28**	-0.08	0.05	0.21	-0.05
BID_{rel}	0.20	0.27	0.12	0.02	**-0.26**	-0.07	0.12	**0.90**	-0.19	1	-0.07	0.10	-0.09	-0.13	-0.03	0.06	-0.13	0.16	0.07
Fr.Sp.	**0.45**	-0.16	-0.16	-0.15	0.09	0.14	-0.19	0.01	**0.30**	-0.07	1	**0.29**	-0.16	**-0.45**	**-0.43**	0.21	**-0.28**	0.19	-0.16
TaP_v	**0.51**	-0.16	-0.01	**0.25**	-0.13	-0.07	-0.12	0.14	0.14	0.10	**0.29**	1	-0.18	**-0.43**	**-0.30**	0.05	-0.22	0.10	**-0.29**
LAT_a	**-0.24**	0.10	**0.36**	0.00	**0.23**	0.07	0.17	-0.04	-0.13	-0.09	-0.16	-0.18	1	**0.37**	**0.30**	0.10	0.10	**-0.25**	0.01
SD CoP	**-0.40**	0.21	0.10	-0.14	**0.23**	-0.08	**0.43**	-0.10	**-0.25**	-0.13	**-0.45**	**-0.43**	**0.37**	1	**0.71**	0.04	0.30	**-0.45**	0.21
D_s	**-0.32**	**0.37**	0.17	-0.02	0.11	-0.23	**0.30**	-0.04	**-0.28**	-0.03	**-0.43**	**-0.30**	**0.30**	**0.71**	1	0.12	0.15	**-0.36**	0.18
H_s	0.11	0.20	0.04	**-0.28**	0.21	0.03	0.10	0.01	-0.08	0.06	0.21	0.05	0.10	0.04	0.12	1	**-0.60**	-0.02	0.10
TP_t	-0.16	0.29	**0.23**	0.12	0.01	-0.11	-0.22	-0.00	0.05	-0.13	**-0.28**	-0.22	0.10	0.30	0.15	**-0.60**	1	-0.29	0.02
H_l	0.23	-0.19	-0.17	-0.12	-0.04	0.00	**-0.37**	0.11	0.21	0.16	0.19	0.10	**-0.25**	**-0.45**	**-0.36**	-0.02	-0.29	1	**0.38**
D_l	-0.09	0.12	0.03	**-0.35**	0.03	-0.05	-0.03	0.10	-0.05	0.07	-0.16	**-0.29**	0.01	0.21	0.18	0.10	0.02	**0.38**	1

H=height; W=weight; Fr.Sp.=frequency of sports; TaP_v=*TaP* variability; LAT_a=*LAT* accuracy; D_s=short-term diffusion coefficient, D_l=long-term diffusion coefficient, H_s=short-term Hurst exponent, H_l=long-term Hurst exponent, TP_t=transition time point
Bold indicates significance ($p \leq 0.05$)
